# A scoping review of COVID-19 modelling studies in Belgium 2020-2024: incorporation of behaviour and lessons learned

**DOI:** 10.1186/s13690-026-01959-3

**Published:** 2026-05-23

**Authors:** Roel Jude Bagaforo, Marie-Cécile Dupas, Steven Abrams, Simon Dellicour, Niel Hens

**Affiliations:** 1 Data Science Institute (DSI), Interuniversity Institute of Biostatistics and statistical Bioinformatics (I-BioStat), Hasselt University, Hasselt, Belgium; 2 Spatial Epidemiology Lab (SpELL), Université Libre de Bruxelles, Brussels, Belgium; 3 Global Health Institute (GHI), Family Medicine and Population Health (FAMPOP), University of Antwerp, Antwerp, Belgium; 4 Department of Microbiology, Immunology and Transplantation, Laboratory for Clinical and Epidemiological Virology, Rega Institute, Katholieke Universiteit Leuven, Leuven, Belgium; 5 Interuniversity Institute of Bioinformatics in Brussels ((IB)²), Université Libre de Bruxelles, Vrije Universiteit Brussel, Brussels, Belgium; 6 Centre for Health Economics Research and Modelling Infectious Diseases (CHERMID), Vaccine & Infectious Disease Institute (VAXINFECTIO), University of Antwerp, Antwerp, Belgium

**Keywords:** SARS-CoV-2, Behaviour, Epidemiological dynamics, Non-pharmaceutical interventions, Pharmaceutical interventions, Mechanistic models, Mathematical models, Statistical models, Ensemble models

## Abstract

**Background:**

The COVID-19 pandemic underscored the importance of integrating human behaviour in infectious disease modelling approaches, yet an in-depth assessment of how behavioural components are incorporated remains limited. We conducted a scoping review of COVID-19 models applied to Belgian data to examine how behavioural dynamics, both voluntary and policy-driven, were represented within model structures. Our aim was to identify current practices, highlight methodological gaps, and provide recommendations for the development of behaviourally integrated epidemiological models.

**Methods:**

Using Scopus and PubMed, we identified 98 studies published between March 2020 and October 2024, describing 105 models in total. Models were classified by model class (mathematical, statistical, or ensemble), objectives, approaches used to incorporate behavioural factors, and types of behaviour data employed.

**Results:**

Behavioural integration was confined to specific modelling contexts, with only half of the 105 models incorporating behavioural components. Mechanistic models, particularly compartmental models, were the most likely to include behavioural features, especially in studies assessing non-pharmaceutical interventions or conducting long-term forecasts and scenario analyses. Behavioural change was most commonly represented through modifications to transmission parameters or contact matrices. These adjustments were frequently informed by social contact surveys or mobility data derived from various sources.

**Conclusions:**

In contrast to previous reviews that focused exclusively on behavioural models, this study evaluates the full landscape of Belgian COVID-19 models, offering a comprehensive perspective on how behavioural representation varies across modelling approaches. Our findings recommend that effective behavioural integration relies on timely, routine, and disaggregated surveillance and behaviour data, alongside the use of flexible mechanistic models.

**Supplementary Information:**

The online version contains supplementary material available at 10.1186/s13690-026-01959-3.

**Table:** 

Text box 1. Contributions to the literature
• This review extends previous work by assessing the full landscape of COVID-19 models applied in Belgium, rather than pre-selecting models already identified as behavioural, enabling a quantification of how prevalent behavioural integration was across modelling approaches.
• It broadens the existing literature by examining both voluntary and policy-driven behaviours and by including statistical and ensemble models alongside mathematical approaches.
• By reviewing all COVID-19 models applied in Belgium, the study shows how often behavioural features were included and under which modelling instances this occurred.
• The paper summarizes key lessons and offers practical recommendations to support effective integration of behaviour in future public health emergencies.

## Background

Human behaviour is a central factor in the spread and mitigation of the circulation of infectious diseases [1]. During the 1918 influenza pandemic, people in the United States [2] and the United Kingdom [3] reduced social contacts as cases increased, influencing transmission patterns. Behavioural shifts were also evident during the COVID-19 pandemic, as individuals adaptively adopted measures such as social distancing, mask use, reducing contacts when symptomatic, avoiding high-risk individuals, and receiving vaccination [4–8]. Behaviour also shapes disease dynamics outside of pandemic contexts. Growing vaccine hesitancy, for example, has contributed to the resurgence of vaccine-preventable diseases such as measles and pertussis [9–11]. In addition to voluntary actions, public health control often relies on behaviour-based interventions, such as isolation, quarantine, and mandatory hygiene measures [12, 13]. Together, these examples show that individual choices and collective behavioural responses play a decisive role in how outbreaks emerge, spread, and can be mitigated.

A growing body of research emphasises the need to explicitly incorporate behavioural factors into the modelling of infectious disease circulation dynamics. Several review papers have examined this topic, particularly focusing on voluntary or self-initiated behaviours and their effects on disease circulation [13–15]. Such behaviours can be represented in models in various ways, for example, through changes in contact rates in response to perceived risk or through adaptive responses driven by information or policy signals. These reviews also stress the importance of validating behavioural models with empirical data and developing frameworks that can capture feedback between human behaviour and epidemic dynamics. For instance, validation can involve comparing model outputs to observed epidemiological trends or survey-based measures of behavioural change. The modelling community within the field of epidemiology increasingly recognises these challenges and continues to advance efforts toward integrated epidemiological–behavioural modelling [16, 17].

The COVID-19 pandemic spurred an unprecedented global expansion of infectious disease modelling. At the same time, it presented a persistent challenge for decision-makers who must meet the urgent demand for timely and actionable insights while working with limited data, constrained resources, and rapidly changing human behaviour [18, 19]. Building on previous reviews, assessing how behavioural dynamics have been incorporated into these models is a crucial step. A fully comprehensive synthesis of this vast modelling landscape, however, is not practically achievable given its breadth. Recent reviews have therefore taken more focused approaches. For instance, Hamilton et al. [20] examined models that included endogenous behaviours, and Lee et al. [21] drew on existing reviews of intervention strategies to identify relevant behavioural components.

Here, our focus is on Belgium, a country that experienced a high COVID-19 burden [22], has a complex multilingual and multi-level governance structure, and hosts an active modelling community that directly supported national and regional decision-making. Rather than examining what behaviours occurred, our focus is on how these behavioural dynamics were incorporated into epidemiological models. Our review considered broader behavioural responses, whether voluntary or policy-driven. We began with the full set of COVID-19 models applied in the Belgian context, allowing us to include both models that incorporated behavioural components and those that did not. In addition, we expanded our scope beyond traditional mathematical models to include statistical and ensemble approaches. This reflects the growing use of these methods during the pandemic for real-time forecasting and policy evaluation, and recognises that behaviour may be represented differently or sometimes overlooked across model types. Briefly, mathematical models describe disease dynamics through explicit equations and/or (micro)simulations, whereas statistical models characterise relationships between variables in a data-driven manner without explicitly representing transmission mechanisms. Ensemble models combine outputs from two or more such models. We examined each model’s objectives and the behaviour data sources it relied on. Based on these findings, we derived key lessons and recommendations and provided an outlook that extends beyond the Belgian context.

## Methods

This scoping review was conducted using the methodological framework proposed by Arksey and O’Malley [23] and adheres to the PRISMA-ScR (Preferred Reporting Items for Systematic Reviews and Meta-Analyses Extension for Scoping Reviews) guidelines [24]. A predefined internal protocol was collaboratively developed by the authors to guide the search strategy, study selection, and data extraction processes, ensuring consistency and transparency throughout the review.

### Search

Following the definition of the inclusion and exclusion criteria and the establishment of the review scope, we searched Scopus and PubMed for peer-reviewed research articles published from March 2020 to 24 October 2024, by which time Belgium had transitioned out of the acute pandemic phase, allowing for a reflection on specific modelling approaches deployed during the crisis and lessons learned thereof. Given the broad nature of the topic of this review and to minimize the risk of excluding relevant studies, we used general search terms (Table 1) and limited our search to both titles and abstracts.

**Table 1:** Search terms used to identify COVID-19 modelling studies in Belgium in Scopusand PubMed (searched up to October 2024)

Search category	Search terms
Geographical scope	Belgium OR Belgian OR Belg*
Disease	COVID-19 OR SARS-CoV-2 OR coronavirus OR COVID OR 2019-nCoV OR nCoV2019
Epidemiological concepts	compartment* OR contact OR immune OR dynamic* OR short-term OR long-term OR scenario OR reproduct* OR mortality OR virulence OR transmiss* OR hospitalisation OR behav* OR spread
Analytical approaches	analys* OR model* OR impact OR effect OR simulat* OR eval*

### Selection

All studies retrieved from the search were screened by two authors (RJB and MCD), who acted as independent primary reviewers. The initial screening was based on titles to assess relevance to the review, followed by the evaluation of abstracts and then full-text articles. In cases of uncertainty regarding a study’s eligibility, disagreements were resolved through discussion and, when necessary, by consulting additional authors (SD and NH) who served as secondary reviewers. Throughout the screening process, we applied the following eligibility criteria:

#### Disease

Although our search terms specified COVID-19 as the disease of interest, we applied an additional criterion to ensure relevance. We included only studies that focused on COVID-19 as the main topic or as one of the primary topics. For example, studies that modelled respiratory syncytial virus (RSV) infection during the pandemic were excluded unless they explicitly addressed co-circulation or interaction with SARS-CoV-2, rather than focusing solely on RSV.

#### Model type

We only included papers that explicitly employed modelling methods to address their research questions. Specifically, we included both mathematical models, such as compartmental models (having, for example, an SEIR structure), agent-based models (also known as individual-based models), and phenomenological models, as well as statistical models, but only when used as a primary modelling framework for spread (e.g., regression, time-series, spatiotemporal, Bayesian, survival, and machine learning models). We excluded studies that used only basic statistical tests, reported descriptive statistics, or applied exploratory data analyses without a formal modelling component, unless they were clearly part of a broader modelling effort.

#### Objective

We included modelling studies that simulate, estimate, or assess the dynamics of disease circulation (e.g., forecasting or estimating epidemiological indicators). These models could, but were not required to, assess the effects of policy interventions (e.g., social distancing, mask use, vaccination, mobility). Studies that focused exclusively on psychological, social, or economic aspects unrelated to population-level disease dynamics were excluded.

#### Study design

We included studies of any design (e.g., cohort, cross-sectional, simulation, surveillance-based, observational, ecological) if they featured a mathematical or statistical modelling component relevant to COVID-19 spread. Cohort and observational studies were included only when modelling was central to the analysis.

#### Population

Only studies involving the circulation of SARS-CoV-2 in humans were included. Studies focusing solely on animals or laboratory simulations without human population dynamics were excluded.

#### Geography

All studies that modelled disease spread in Belgium were eligible. This encompassed multi-country studies, simulation studies contextualised to a Belgian setting, case studies using Belgian data, as well as studies conducted in smaller communities such as provinces or specific institutions within Belgium.

#### Original research

Review articles, editorials, comments, preprints, and letters were excluded.

#### Language

Only articles published in English were included.

To ensure the completeness of our search and to identify non-published operational models, we cross-checked our identified records with the RESTORE consortium [25]. RESTORE is a Belgian inter-university collaboration, comprising research groups from universities across Flanders (Ghent University, KU Leuven, University of Antwerp, Hasselt University), Wallonia (University of Namur), and Brussels (Vrije Universiteit Brussel, Université Libre de Bruxelles), established in May 2020 to produce ensemble scenario analyses for SARS-CoV-2 spread.

**Fig. 1:**
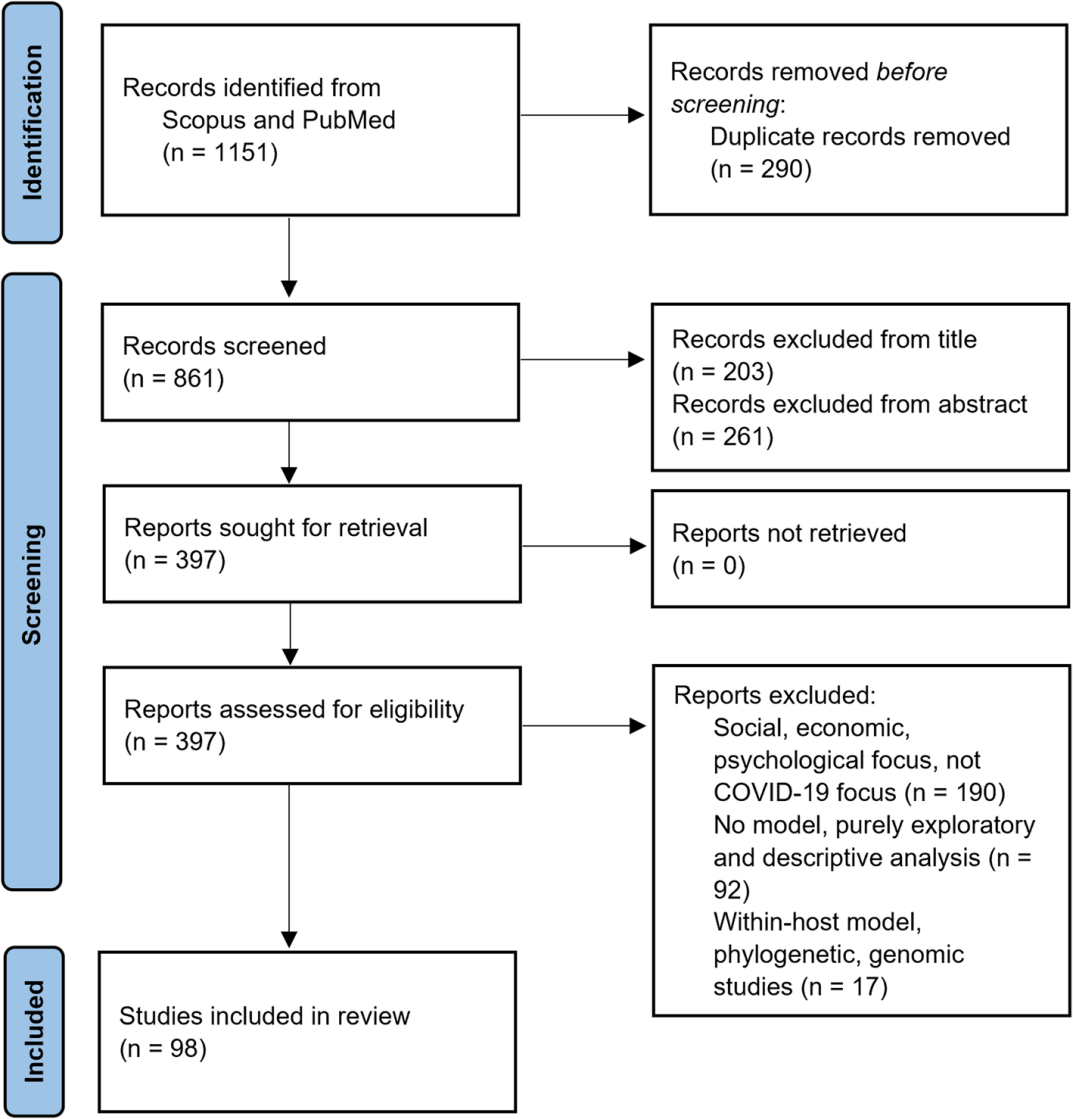
PRISMA flowchart of the search and selection process for COVID-19 modelling studies in Belgium (searched up to October 2024). PRISMA: Preferred Reporting Items for Systematic Reviews and Meta-Analyses

The results of the search and selection process are presented in Fig. 1 and discussed in detail in Search results section.

### Data extraction

For each eligible article, RJB and MCD independently extracted the following data from the full text: (i) modelling methodology; (ii) study objectives; (iii) geographical scope and boundaries; (iv) study period and publication date; (v) limitations as reported by study authors; (vi) input data/metrics; (vii) output data/metrics; (viii) model validation approach; (ix) key conclusions and recommendations; (x) integration of vaccination dynamics; (xi) model calibration process; and (xii) behavioural components, including how they were modelled and parameterised. Additional relevant details were recorded in free-form fields when applicable. Any discrepancies or uncertainties during extraction were resolved through discussion between primary and secondary reviewers to reach consensus.

Since some studies employed multiple distinct models (e.g., compartmental models alongside agent-based models), we separated and analysed them by model class to enable comparison of methodological differences. Consequently, the final selection contained more models than papers, reflecting this disaggregation. In addition, although several studies appeared to be based or developed from the same underlying model framework, the level of detail reported was not always sufficient to systematically trace model reuse. Therefore, each publication was treated as a distinct modelling study when it addressed a different research objective and/or implemented modifications to the model. This approach aligns with the aim of the scoping review to map how behavioural components were incorporated into COVID-19 models across studies, rather than to catalogue unique model frameworks, and allowed us to capture variations and developments in behavioural assumptions, data sources, and implementation over time.

### Objectives

The primary objective of this review is to map how behaviour was integrated into COVID-19 models applied in the Belgian context. To do so, we began with the full set of identified models, regardless of whether they incorporated behavioural components, allowing us to assess the prevalence of behavioural integration across different modelling contexts. We broadly defined behaviour as any individual or collective response to the pandemic, whether voluntary or policy-driven. This includes actions stemming from risk perception, such as reducing contacts out of fear of infection, as well as responses to imposed interventions, such as complying with mandated isolation. This definition is intentionally broader than those used in previous works, such as by Funk et al. [13] and Verelst et. al. [14], which focused exclusively on self-initiated and voluntary behaviours. For models identified as having a behavioural component, we further classified how behaviour change was represented, following the framework proposed by Funk et al. [13]. This framework outlines three categories: (1) behaviour that modifies model parameters, (2) behaviour that alters disease states, and (3) behaviour that changes the contact structure.

Because infectious disease models were long regarded as primarily conceptual, integrating statistical methods and empirical data was essential to enable model fitting to real-world observations. This gap is particularly evident in behavioural epidemic models, where only a limited number have been calibrated using empirical behaviour data [16]. Accordingly, in addition to mapping how behaviour has been conceptualised, we examined the extent to which models with behavioural components incorporated empirical behaviour data during calibration. Specifically, we focused on how the model parameters were informed and estimated, the types of data used, and the inferential paradigms employed.

## Results

### Search results

Our initial search in Scopus and PubMed yielded 1,151 papers (Fig. 1). After removing 290 duplicates, we screened 861 records based on title, abstract, and full-text. To minimise the risk of excluding relevant studies, we adopted a lenient approach during title/abstract screening, as key methodological details were often only discernible in the full text. Following this, 397 full-text articles were assessed for eligibility, of which 98 met our inclusion criteria. The majority of exclusions were due to studies focusing on non-epidemic aspects, such as social, economic, or psychological impacts (particularly mental health and societal effects). Additional exclusions involved papers with purely descriptive analyses, lacking predictive or explanatory models, or molecular epidemiology studies. From the 98 included papers, we extracted data on 105 models. Cross-referencing our results with the models from the RESTORE consortium [25] confirmed that our review captured all but one agent-based model. We further noted that most RESTORE models addressed objectives similar to those identified in our database search.

### Modelling landscape in Belgium

#### Model classes

We classified models into three main classes: mathematical, statistical, and ensemble models. Here, the term mathematical models follows the convention commonly adopted in the infectious disease modelling literature to distinguish equation-based approaches from purely data-driven statistical ones, rather than referring to the broader mathematical nature of all modelling frameworks. Mathematical models include compartmental models, which are population-based and derived from the basic SIR framework (Kermack and McKendrick, [26]) and its extensions (e.g., Keeling and Rohani [27], Vynnycky and White [28]); meta-population models, which extend compartmental models by incorporating disaggregated geographic patches linked through movement or migration (e.g., Ball et al. [29]); agent-based models, which simulate interactions at the individual level (e.g., Willem et al. [30]); and phenomenological models, which describe disease dynamics through fitting mathematical functions to the observed shape of the epidemic curves without explicit mechanistic assumptions (e.g., logistic and diffusion model). Statistical models are categorised into mean regression models (focusing on relationships between variables), spatial statistical models (accounting for spatial autocorrelation and geographic variation), time-series models (analysing temporal trends), machine learning models (using data-driven predictive algorithms), and other models that do not fit into these groups. Ensemble models combine two or more mathematical and/or statistical models to generate a unified output. For detailed definitions and example models, please refer to the Supplementary Material (Section S2).

Among the 105 models reviewed, mathematical and statistical models were used in nearly equal proportions, with 57 (54.29%) and 44 (41.90%) models, respectively (Table 2). Only 4 models (3.81%) were classified as ensemble models. Within the mathematical model class, the majority were compartmental models (61.40%; 35 of 57), followed by phenomenological models (21.05%; 12 of 57). Among the statistical models, most were regression-based (40.91%; 18 of 44). Beyond this model classification, models can also be grouped into mechanistic and non-mechanistic groups. Mechanistic models explicitly represent the underlying biological processes, while non-mechanistic models are typically data-driven and do not model these mechanisms directly. According to this classification, all mathematical models that are not phenomenological in nature, namely compartmental, agent-based, and metapopulation models, are considered mechanistic and represent 42.86% (49 of 105) of all models.

**Table 2:** Summary^a^ of the characteristics of COVID-19 models identified in Belgium (2020–2024) included in the scoping review

Characteristics	Mathematical models				Statistical models					Ensemble models	Total
	Compartmental models	Phenomenological models	Agent/Individual-based models	Metapopulation models	Regression models	Spatial models	Time-series models	ML^b^ Models	Others		
Objectives^+^											
Impact of NPI^c^	18 (51.43%)	2 (16.67%)	3 (37.50%)	2 (100.00%)	7 (38.89%)	3 (50.00%)	1 (25.00%)	0 (0.00%)	3 (25.00%)	0 (0.00%)	39 (37.14%)
Impact of PI^d^	10 (28.57%)	1 (8.33%)	1 (12.50%)	1 (50.00%)	2 (11.11%)	1 (16.67%)	1 (25.00%)	0 (0.00%)	3 (25.00%)	0 (0.00%)	20 (19.05%)
Vaccination	7 (20.00%)	1 (8.33%)	1 (12.50%)	1 (50.00%)	1 (5.56%)	1 (16.67%)	1 (25.00%)	0 (0.00%)	3 (25.00%)	0 (0.00%)	16 (15.24%)
Impact	0 (0.00%)	1 (8.33%)	1 (12.50%)	1 (50.00%)	1 (5.56%)	1 (16.67%)	1 (25.00%)	0 (0.00%)	3 (25.00%)	0 (0.00%)	9 (8.58%)
Distribution/Allocation/Prioritisation	5 (14.29%)	0 (0.00%)	0 (0.00%)	0 (0.00%)	0 (0.00%)	0 (0.00%)	0 (0.00%)	0 (0.00%)	0 (0.00%)	0 (0.00%)	5 (4.76%)
Coverage/Threshold/Characteristics	2 (5.71%)	0 (0.00%)	0 (0.00%)	0 (0.00%)	0 (0.00%)	0 (0.00%)	0 (0.00%)	0 (0.00%)	0 (0.00%)	0 (0.00%)	2 (1.90%)
Short forecasting/scenario	6 (17.14%)	6 (50.00%)	0 (0.00%)	0 (0.00%)	2 (11.11%)	0 (0.00%)	2 (50.00%)	1 (25.00%)	3 (25.00%)	1 (25.00%)	21 (20.00%)
Long forecasting/scenario	12 (34.29%)	4 (33.33%)	3 (37.50%)	2 (100.00%)	0 (0.00%)	0 (0.00%)	0 (0.00%)	2 (50.00%)	0 (0.00%)	1 (25.00%)	24 (22.86%)
Disease metrics	22 (62.86%)	4 (33.33%)	5 (62.50%)	0 (0.00%)	12 (66.67%)	5 (83.33%)	1 (25.00%)	1 (25.00%)	8 (66.67%)	3 (75.00%)	61 (58.10%)
Scope/Boundary											
Multi-country	22 (62.86%)	10 (83.33%)	2 (25.00%)	0 (0.00%)	12 (66.67%)	0 (0.00%)	4 (100.00%)	2 (50.00%)	5 (41.67%)	4 (100.00%)	61 (58.10%)
National	10 (28.57%)	2 (16.67%)	6 (75.00%)	2 (100.00%)	6 (33.33%)	6 (100.00%)	0 (0.00%)	2 (50.00%)	7 (58.33%)	0 (0.00%)	41 (39.05%)
Subnational	3 (8.57%)	0 (0.00%)	0 (0.00%)	0 (0.00%)	0 (0.00%)	0 (0.00%)	0 (0.00%)	0 (0.00%)	0 (0.00%)	0 (0.00%)	3 (2.86%)
Granularity											
National level	31 (88.57%)	11 (91.67%)	0 (0.00%)	0 (0.00%)	14 (77.78%)	0 (0.00%)	4 (100.00%)	3 (75.00%)	5 (41.67%)	4 (100.00%)	72 (68.57%)
Subnational level	2 (5.71%)	0 (0.00%)	0 (0.00%)	2 (100.00%)	1 (5.56%)	6 (100.00%)	0 (0.00%)	0 (0.00%)	3 (25.00%)	0 (0.00%)	14 (13.33%)
Micro-level (hospitals, households, etc.)	2 (5.71%)	1 (8.33%)	2 (25.00%)	0 (0.00%)	0 (0.00%)	0 (0.00%)	0 (0.00%)	1 (25.00%)	0 (0.00%)	0 (0.00%)	6 (5.71%)
Individual level	0 (0.00%)	0 (0.00%)	6 (75.00%)	0 (0.00%)	3 (16.67%)	0 (0.00%)	0 (0.00%)	0 (0.00%)	4 (33.33%)	0 (0.00%)	13 (12.38%)
Age-structured	9 (25.71%)	0 (0.00%)	7 (87.50%)	2 (100.00%)	4 (22.22%)	6 (100.00%)	0 (0.00%)	0 (0.00%)	5 (41.67%)	0 (0.00%)	33 (31.43%)
Spatially structured	1 (2.86%)	0 (0.00%)	0 (0.00%)	2 (100.00%)	1 (5.56%)	6 (100.00%)	0 (0.00%)	0 (0.00%)	1 (8.33%)	0 (0.00%)	11 (10.48%)
Stochastic^e^	11 (31.43%)	-	8 (100.00%)	2 (100.00%)	-	-	-	-	-	-	22 (20.95%)
Parameter/s estimated/calibrated	24 (68.57%)	2 (16.67%)	2 (25.00%)	2 (100.00%)	18 (100.00%)	6 (100.00%)	4 (100.00%)	4 (100.00%)	12 (100.00%)	-	74 (70.48%)
Frequentist inference	13 (37.14%)	0 (0.00%)	1 (12.50%)	0 (0.00%)	18 (100.00%)	2 (33.33%)	4 (100.00%)	0 (0.00%)	4 (33.33%)	-	42 (40.00%)
Bayesian inference	11 (31.43%)	0 (0.00%)	1 (12.50%)	2 (100.00%)	0 (0.00%)	4 (66.67%)	0	0 (0.00%)	8 (66.67%)	-	27 (25.71%)
Others (e.g., mathematical derivations)	0 (0.00%)	2 (16.67%)	0 (0.00%)	0 (0.00%)	0 (0.00%)	0 (0.00%)	0 (0.00%)	4 (100.00%)	0 (0.00%)	-	6 (5.71%)
With behaviour component	25 (71.43%)	2 (16.67%)	8 (100.00%)	2 (100.00%)	7 (38.89%)	2 (33.33%)	0 (0.00%)	0 (0.00%)	2 (16.67%)	0 (0.00%)	48 (45.71%)
Behavioural change^+^											
Change in parameter	18 (51.43%)	1 (8.33%)	0 (0.00%)	0 (0.00%)	-	-	-	-	-	0 (0.00%)	19 (18.10%)
Change in disease state	5 (14.29%)	1 (8.33%)	1 (12.50%)	1 (50.00%)	-	-	-	-	-	0 (0.00%)	8 (7.62%)
Change in contact structure	10 (28.57%)	0 (0.00%)	7 (87.50%)	2 (100.00%)	-	-	-	-	-	0 (0.00%)	19 (18.10%)
Behaviour data											
Mobility	2 (5.71%)	0 (0.00%)	0 (0.00%)	0 (0.00%)	4 (22.22%)	2 (33.33%)	0 (0.00%)	0 (0.00%)	1 (8.33%)	0 (0.00%)	9 (8.57%)
Social contacts	8 (22.86%)	1 (8.33%)	7 (87.50%)	0 (0.00%)	0 (0.00%)	0 (0.00%)	0 (0.00%)	0 (0.00%)	1 (8.33%)	0 (0.00%)	17 (16.19%)
Both	2 (5.71%)	0 (0.00%)	0 (0.00%)	2 (100.00%)	2 (11.11%)	0 (0.00%)	0 (0.00%)	0 (0.00%)	0 (0.00%)	0 (0.00%)	6 (5.71%)
Total	35	12	8	2	18	6	4	4	12	4	$N=105$

^a^Presented are frequencies with columnwise proportions inside parentheses. Proportions are calculated relative to the total number of models within each model class (see Total row)

^b^Machine Learning, ^c^Non-pharmaceutical Interventions, ^d^Pharmaceutical Interventions

^e^Only relevant for mechanistic models (stochastic or deterministic)

^+^These characteristics are *not* mutually exclusive; models may fall into multiple categories under this characteristic

Most models categorised as ensemble models lacked information on their source models. We did not delve deeper to retrieve information and relied on the main text

Behavioural change classification was not applicable for statistical models. Most models included behaviour or behavioural change as covariate(s)

#### Model objectives

We identified and categorised each model’s objectives (see Section S3 in the Supplementary Material for more details). These include assessing the impact of non-pharmaceutical interventions (NPIs; e.g., lockdowns, social distancing, quarantine), pharmaceutical interventions (e.g., vaccination, antivirals, testing), generating forecasts or scenario analyses (classified as short-term if under one month, and long-term otherwise), and estimating key disease metrics (e.g., reproduction number, attack rate, peak timings, excess mortality). We also highlighted vaccination-related objectives and identified whether the models were used to assess the impact of vaccination, or to inform distribution, allocation, and prioritisation strategies, and/or to determine vaccine coverage thresholds and other vaccine characteristics. These classifications are not mutually exclusive, meaning a single model can serve multiple objectives.

More than half of the models (58.10%; 61 of 105) were used to estimate disease metrics, primarily the effective reproduction number. Other objective categories, including assessing the impact of non-pharmaceutical and pharmaceutical interventions, and short- and long-term forecasting/scenario analyses, were also well represented, with around 20% of models addressing each. Among the model classes, compartmental models stood out as the most flexible, being applied across a wide range of objectives. Phenomenological models were used primarily to forecast or estimate specific disease metrics. Although other mathematical model types addressed multiple objectives, their overall contribution was limited due to the smaller number of models in these classes. For statistical models, regression, spatial, and time-series approaches were primarily used to assess the impact of interventions and estimate disease metrics, while machine learning models were predominantly used for forecasting.

#### Model characteristics

We also examined additional model characteristics for further insight. About three-fifths (61 of 105) of the models were multi-country, meaning the same model framework was applied to describe disease spread in multiple countries within the study. This was especially common among compartmental (62.86%; 22 of 35) and regression models (66.67%; 12 of 18). In contrast, agent-based and spatial models were predominantly used in single-country settings (6 of 8 and 6 of 6, respectively). Regarding model granularity, defined here as the finest unit of analysis represented in the model (as distinct from scope, which refers to the geographic coverage), nearly 70% (72 of 105) of the models focused on the national population level. Most compartmental and phenomenological models were deployed at the national level, whereas agent-based models typically operated at finer, more granular levels. Statistical models showed greater variability in the level of granularity considered. Relatively few models incorporated age structure or spatial structure, indicating that these sources of heterogeneity were often ignored. We also found that all agent-based and meta-population models were stochastic, whereas only about one-third of compartmental models were (i.e., 11 models). Of the 105 models, 73 reported parameter estimation or calibration, with 38 using frequentist and 28 using Bayesian inference. The remaining 7 models relied on alternative calibration approaches, such as analytical derivations, and algorithmic or machine learning-based procedures (e.g., test-train splits and cross-validation).

### Behaviour

Having outlined the overall landscape of models used in a Belgian context, we now focus on the role of behavioural processes within these models. We first describe the extent to which behavioural components are included across model classes and objectives (Behaviour and behaviour change section). In Integrating behaviour data section, we then examine how behaviour is represented and operationalised, highlighting common approaches and the data used to inform them.

#### Behaviour and behaviour change

**Fig. 2:**
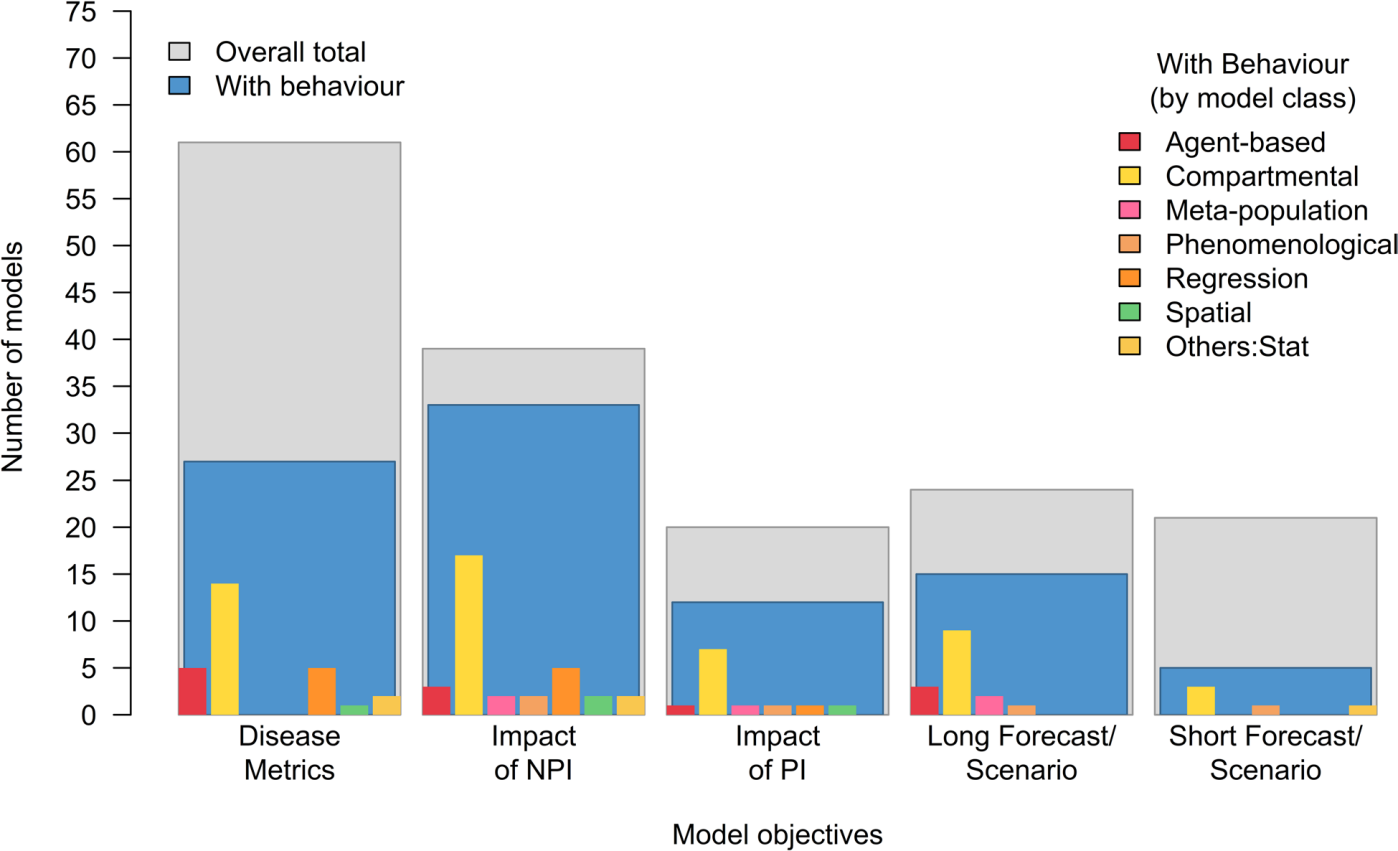
Number of COVID-19 models identified in Belgium (2020–2024) per objective category. For each model objective (*x*-axis), the height of the grey bar represents the total number of identified models, while the height of the blue bar represents the total number of models incorporating behavioural components (on the *y*-axis). The coloured bars show the breakdown of models with behavioural components by model class. Note that objective categories are not mutually exclusive, i.e., a single model may address multiple objectives simultaneously

Out of the 105 models, about half (*n* = 48; 45.71%) incorporated behavioural features. Behavioural components were more commonly integrated into mathematical models than in statistical ones, with 65% (37 of 57) of mathematical models including behaviour, compared to only 25% (11 of 44) of statistical models. We could not determine whether ensemble models included behaviour because we were unable to retrieve the details of their individual component models. As depicted in Fig. 2, models used to assess the impact of NPIs (33 out of 39 models) and to generate longer forecasts/scenario analyses (15 of 23 models) were more likely to include a behavioural component compared to those addressing the impact of pharmaceutical interventions and generating short forecasts/scenarios. Again, mathematical models, particularly compartmental, agent-based, and meta-population models, were the most commonly used model classes that incorporate behaviour across all objective categories. Among these, compartmental models were the most prominent.

All models we found with behavioural components incorporated behaviour dynamically (i.e., behaviour change over time; see Section S4 in Supplementary Material for definition), recognising that human responses to the spread of COVID-19 were non-static. As such, we classified how behaviour change was represented in the models based on the framework proposed by Funk et al. [13], which outlines three categories: (1) behaviour that modifies model parameters, (2) behaviour that alters disease states, and (3) behaviour that changes the contact structure. These categories are not mutually exclusive, so a single model may exhibit more than one. This classification was applied to mathematical models as their structure supports such distinctions. For statistical models, this classification was not applied because behaviour change was generally included as a time-varying covariate or regressor, rendering the aforementioned classifications meaningless.

The most common representations of behavioural change involved modifications to model parameters or contact structures (Table 3). A typical approach was to adjust the transmission rate in response to NPIs. In some models, these adjustments were done based on defining change points according to the NPI timeline [31–36]. Other models used scaling factors based on a mobility index [37–39] or on a stringency index (a composite score quantifying the strictness of government interventions over time) [40] (see Integrating behaviour data section for a description of mobility data and indexes). Several models introduced compliance or step functions to reflect hesitation or delayed adherence to interventions, acknowledging that people may not comply immediately [22, 41–44]. In terms of contact structure, several models, particularly those with age stratification, used social contact matrices to represent interactions among age groups, thereby relaxing the assumption of homogeneous mixing. Some models even differentiated contact patterns based on the presence and development of symptoms, using separate matrices for symptomatic and asymptomatic individuals to reflect differences in behaviour [22, 41–45]. It should be noted that these contact matrices directly influence the transmission parameter, placing them in the category of parameter modification. However, we treated these models separately because they incorporated explicit contact structures. The most common representations of contact structure were based on age-specific social contact data (see Integrating behaviour data section for a description of social contact data). Only a small number of models included behaviour-driven changes in disease states. Most of these introduced a compartment for vaccinated individuals, reflecting the voluntary nature of COVID-19 vaccination, whereby individuals transitioned to this vaccinated state. The majority were informed by observed vaccine uptake information.

**Table 3:** Behavioural change classifications and behaviour data sources used in COVID-19 models identified in Belgium (2020–2024)

**Behavioural change classification**	
**Classification**	**Publications**
Modifies parameter	Abrams et al. [41]; Šušteršič et al. [31]; Rémond and Rémond [32]; Dashtbali and Mirzaie [46]; Yin et al. [37]; Chin et al. [38]; Qiu et al. [39]; Cellès et al. [40]; Reymond et al. [42]; Kozyreff [33]; Post et al. [34]; Brugnago et al. [35]; Kohanovski et al. [36]; Peirlinck et al. [47]; Willem et al. [43]; Devleesschauwer et al. [22]; Lmater et al. [48]; Dashtbali [49]; Molenberghs et al. [44]
Changes the contact structure	Abrams et al. [41]; Alleman et al. [50]; Alleman et al. [51]; Coletti et al. [52]; Franco [53]; Willem et al. [54]; Angeli et al. [55]; Kuylen et al. [56]; Libin et al. [45]; Reymond et al. [42]; Møgelmose et al. [57]; Willem et al. [43]; Devleesschauwer et al. [22]; Loedy et al. [58]; Møgelmose et al. [59]; Arnst et al. [60]; Molenberghs et al. [44]^a^; Yin et al. [61]
Alters disease states	Alleman et al. [50]; Dashtbali and Mirzaie [46]; Hazard-Valdés and Montero [62]; Matrajt et al. [63]; Yin et al. [37]; Willem et al. [43]; De Meijere et al. [64]; Denoël et al. [65]
**Behaviour data**	
**Behaviour data**	**Publications**
Social contact data only	Abrams et al. [41]; Franco [53]; Willem et al. [54]; Angeli et al. [55]; Kuylen et al. [56]; Libin et al. [45]; Reymond et al. [42]; Møgelmose et al. [57]; Willem et al. [43]; Devleesschauwer et al. [22]; Loedy et al. [58]; Møgelmose et al. [59]; Loedy et al. [66]; Molenberghs et al. [44]^a^; Yin et al. [61]; De Meijere et al. [64]
Mobility data only	Yin et al. [37]; Chin et al. [38]; Chin et al. [67]; Rollier et al. [68]; Jamison et al. [69]; Pana et al. [70]; Nguyen et al. [71]; Ensoy-Musoro et al. [72]; Habib et al. [73]
Both (mobility + social contact data)	Alleman et al. [50]; Alleman et al. [51]; Coletti et al. [52]; Post et al. [34]; Lajot et al. [74]; Wambua et al. [75]

^a^Paper includes two models or the application of two distinct models

In statistical models, behavioural components were incorporated as explanatory variables rather than as structural features. Specifically, these models capture associations between behavioural proxies, such as mobility indices, stringency scores, or social contact rates, and epidemiological outcomes (e.g., case counts or mortality), without explicitly modelling the underlying mechanisms driving these relationships. Calibration was performed using standard statistical inference, treating behaviour as an external input rather than a dynamic process.

#### Integrating behaviour data

The most commonly used data types included intervention timelines, stringency indexes, social contact surveys, and mobility data. Intervention timelines provide dates and details for implementing policies such as lockdowns or mask mandates, while stringency indexes quantify the strictness of these interventions over time, combining multiple policy measures into a single score. Social contact surveys collect information on the frequency and nature of interpersonal interactions, offering insight into changes in social behaviour. Mobility data, often derived from mobile devices, track population movement patterns and changes in activity levels. It is important to note that stringency indexes reflect the intended strictness of interventions but do not necessarily capture how people actually respond to them. In contrast, social contact surveys and mobility data provide more direct evidence of observed behavioural responses, thereby reflecting real-world behaviour more closely. Of the 48 models with behavioural components, 9 used mobility data, 17 used social contact data, and 6 incorporated both (Table 3). Most of the remaining models with behavioural components that did not use behaviour data (16 out of 48 models) applied time-dependent functions tied to specific dates when policies were implemented (e.g., assuming a certain level of behaviour change occurred after a lockdown announcement).

Most of the mobility data came from Google’s Community Mobility Reports [76], which provide daily indices of movement trends across various locations using anonymised location data from mobile devices. Some models used similar mobility data from telecommunications companies with greater spatial disaggregation. On the other end, a variety of social contact data sources were used across the models. During the early phase of the pandemic, projected contact matrices [77] and historical surveys were commonly relied upon, including the POLYMOD study [78], the 2010–2011 Belgian social contact survey [79], and the 2009 H1N1 influenza pandemic contact survey in London [80]. These pre-pandemic contact data were used as proxies for baseline contact patterns. As the pandemic progressed, the CoMix survey [81], a longitudinal social contact survey conducted during 2020–2022, became an important data source, especially for studies focused on the mid to late phases of the pandemic. Many of these contact data were obtained via the SOCRATES tool [82, 83], an open platform for sharing and analysing social contact survey data, or through the website socialcontactdata.org [84].

These behaviour data were incorporated into the models in various ways. For statistical models that used mobility data, mobility served as a covariate or control variable to represent behaviour change [67–73]. Conversely, in mathematical models, mobility was used as a scaling factor applied to the transmission parameter [37, 38]. The models that used only social contact data were mainly mathematical. These models typically integrated the contact data by applying the social contact hypothesis [85], which assumes that transmission is directly proportional to social contacts (see Section S5 of the Supplementary Material for more details). Many of these models used social contact matrices to differentiate behaviour of individuals based on their disease state (e.g., asymptomatic or in isolation) [22, 41, 43–45, 53–55, 64]. Also, most of these models used social contact data to represent longitudinal behaviour changes. For example, Abrams et al. [41] started with pre-pandemic contact matrices and adjusted different contact matrices to resemble exit strategies and to quantify their impact on the epidemic trends, while Willem et al. [43] relied on the longitudinal CoMix survey as these data became available later during the course of the pandemic. Other models, such as those by Alleman et al. [51] and Alleman et al. [50], scaled pre-pandemic contact matrices using mobility data. Finally, in metapopulation models, mobility data were used to parameterise movement between spatial units, allowing models to explicitly capture connectivity between regions and simulate the spatial spread of infection across locations [50, 52].

## Discussion

Human behaviour plays a crucial role in the spread and mitigation of infectious diseases. Mitigation measures such as social distancing, mask-wearing, and changing mobility patterns can significantly affect the course of an outbreak. To improve the accuracy and usefulness of disease models, it is important to include these behavioural responses, especially during fast-changing situations like the COVID-19 pandemic. With this in mind, we conducted a scoping review of COVID-19 models developed for the Belgian setting, examining how they accounted for behavioural factors. We screened a total of 1,151 papers from PubMed and Scopus and selected 98 studies describing a total of 105 models. We analysed the general features of these models and how they included behavioural components and behaviour data.

Mechanistic models were the most commonly used and also the most likely to incorporate behavioural components (Table 4; Lesson 1). Their structural flexibility enabled the explicit representation of behavioural responses and the evaluation of NPIs on transmission dynamics. In contrast, statistical and phenomenological models remained largely data-driven, treating behavioural variables as exogenous explanatory predictors rather than dynamic processes. Among mechanistic models, compartmental frameworks were the most prevalent and were applied across a wide range of settings (e.g., multiple countries and population segments), likely reflecting the substantial computational and calibration demands of agent-based and metapopulation approaches [29, 30]. The lower prevalence of behavioural integration in statistical models is partly structural; these models are designed to estimate associations between variables rather than simulate dynamic processes. Consequently, behaviour is typically included as an exogenous covariate, highlighting a fundamental difference from mechanistic approaches.

**Table 4:** Lessons learned and recommendations for the incorporation of behaviour derived from a scoping review of COVID-19 modelling studies in Belgium (2020–2024)

	Lesson	Recommendation
1	Mechanistic modelling frameworks are currently the primary vehicles for integrating behavioural dynamics.	Future modelling efforts should therefore prioritise and advance the development and use of flexible, readily calibratable mechanistic models that can accommodate dynamic and complex behavioural representations, particularly in rapidly evolving epidemic contexts.
2	Integration of behaviour hinged and varied according to model objectives.	Model utility should be explicitly defined at the outset of model development, with behavioural components selected in direct alignment with the model’s intended purpose. Sensitivity analyses of behavioural assumptions should be routinely conducted to assess their influence on model outputs, and ensemble approaches may be considered to capture structural and behavioural uncertainty.
3	Integrated behaviour data were limited by availability and compatibility.	Sustained collection of model-compatible behaviour data beyond crisis periods is essential to reduce reliance on ad hoc or proxy-based inputs. Data collection initiatives should be co-designed with modelling needs in mind, enabling closer alignment between data structures and model requirements and helping to narrow persistent model–data gaps.
4	Behavioural heterogeneity was constrained by behaviour and surveillance data	Investment in more granular, diverse, and complementary behaviour and surveillance data is needed to better represent population heterogeneity. Modelling studies should contribute to this process by explicitly investigating and reporting the levels of data granularity and characteristics required for effective behavioural representation, thereby informing future data collection strategies.
5	Behavioural components were predominantly incorporated exogenously.	Further methodological development is needed to design and empirically validate endogenous behavioural representations grounded in established health behaviour or psychological theories. Model scrutiny, through transparent documentation of assumptions, sensitivity analyses, and collaborative review, should be considered essential to support model credibility.

The integration of behaviour was highly contingent on the model’s objective (Table 4; Lesson 2). Behavioural components were prioritised in studies examining the impact of NPIs or long-term forecasts/scenarios, but were frequently omitted in short-term forecasting. This pattern suggests that behaviour was not treated as an intrinsic driver of epidemiological dynamics, but rather as a factor considered only when the modelling objective demanded it. This distinction is not merely descriptive. It reflects a broader principle that the decision to include behavioural components should be guided by the model’s intended purpose. For short-term forecasting under stable conditions, simpler models without explicit behavioural components may perform adequately, while behavioural integration becomes more critical when evaluating interventions or projecting over longer time horizons where human responses are likely to alter. Importantly, however, the extent to which behaviour can be integrated is also strongly dependent on a model’s capacity to address a given objective (e.g., machine learning models designed primarily for forecasting rather than for evaluating intervention effects). This reinforces the importance of clearly defining model purpose upfront, aligning behavioural assumptions accordingly, and conducting sensitivity analyses to assess their impact on model outputs. In this regard, while behavioural change in these models was typically represented as adjustments to contact structures or transmission parameters, recent evidence suggests that incorporating behaviour-altering disease states, such as an explicit risk-averse state, can enhance model performance [86].

Among the models we reviewed, social contact and mobility data were the most commonly used forms of behaviour data. Their frequent use likely reflects both their broad availability and their compatibility with modelling frameworks that can incorporate such data (Table 4; Lesson 3). These data types were drawn from diverse sources. Social contact data, in particular, were typically obtained from surveys conducted before or during the pandemic, allowing models to be parametrised by constructing baseline and intervention-period contact matrices [41, 43, 51]. Together, these findings highlight the importance of conducting social contact surveys and systematically collecting behaviour data in both peacetime and crisis periods. Anonymised mobility data were sourced either from Google or from telecommunication companies, each offering different levels of quality and granularity. As a result, models were often adapted to fit the available data, influencing spatial domain choices [50, 52, 72] and model parameterisation [37, 68, 71]. This points to a broader issue in epidemic modelling, namely the frequent mismatch between the data required by models and the data actually available. It also emphasises the importance of developing more adaptable modelling frameworks capable of handling diverse and sometimes imperfect data sources. At the same time, it presents an opportunity to explore model-compatible behaviour data beyond the aforementioned social contact and mobility measures, for example, by taking advantage of the abundance of big data or linking multiple data sources.

We also examined how models addressed heterogeneity in behaviour, capturing differences by age group, location, and health state. Age-based differences in transmission were often captured using social contact data, showing how different age groups interact putting those with more frequent contacts at higher risk of infection, while spatial differences were modelled using mobility data to represent travel patterns. Some compartmental models also adjusted contact behaviour based on disease state, such as whether individuals were symptomatic, asymptomatic, or in isolation, acknowledging how health status influences behaviour. Although these approaches aim to account for behavioural diversity, they often overlook other important factors, such as socioeconomic status, gender, and risk perception [87–90]. Integrating these additional layers of heterogeneity, however, requires equally detailed surveillance data, which are often unavailable and thereby limit how comprehensively behaviour can be represented (Table 4; Lesson 4). Furthermore, model choice matters in representing more detailed heterogeneity. In particular, agent-based models can more naturally reflect individual-level heterogeneity arising from variation in individual behaviour and behaviour change.

Whilst several models incorporated data-driven behavioural components, none of these explicitly drew from adherence studies, risk perception surveys, or broader health behaviour theories (Table 4; Lesson 5). Instead, changes in contact and mobility patterns were commonly used as proxies for behavioural responses, implicitly capturing dynamics such as compliance, intervention fatigue, or reactions to the growing spread [5, 91]. Similarly, when modelling the impact of vaccination, most studies relied on vaccination uptake data without explicitly incorporating the social or psychological factors that shape vaccine decision-making or potential post-vaccination risk compensation leading to reduced adherence to NPIs [92–94]. These approaches allow for some behavioural representation, but blur the line between voluntary adaptation and behaviour driven by interventions. Moreover, the relationship between social contact and mobility remains unclear [95], making it difficult to interpret which signals reflect which aspects of behaviour. Models grounded in behavioural theory, such as feedback-based or game-theoretic approaches, offer the potential to better reflect dynamic, endogenous behaviour but were notably absent from our review. Evidence suggests that endogenous models may outperform exogenous ones [20, 86, 96], but no definitive consensus has emerged. Routine model scrutiny, systematic cross-validation of different models among modellers, and possibly the use of ensemble approaches, therefore, remain important. It is also worth noting that greater behavioural detail may not always improve generalisability. Models relying on context-specific behavioural data may fit local dynamics well but be difficult to transfer, highlighting a trade-off that should be considered during model development.

Although focused on studies dedicated to or including Belgium, our study takes a broader perspective by including both self-initiated behaviours and general behavioural responses, whether voluntary or policy-driven. Unlike previous reviews, which typically examined only models already identified as incorporating behavioural components, we began with the full set of models and then determined which explicitly included behavioural components. Consequently, we identified the proportion of models integrating behavioural components and found more models than previous reviews [13–15, 20] (with some reviews dating back to the pre-COVID era). While it would be of interest to compare the performance of models that incorporated behaviour with those that did not, such an analysis would require common modelling conditions and a formal systematic review. Furthermore, given publication delays, some relevant modelling studies conducted during the pandemic may have appeared after our October 2024 search cutoff and are therefore not captured in this review.

Our findings partly align with those of Lee et al. [21], particularly regarding the reliance on mobility data and the predominant use of mechanistic models. Distinctively, our review further explores how behaviour was represented across different modelling objectives, the types of behaviour data used, and how these were integrated into both mathematical and statistical frameworks. For instance, while Lee et al. [21] highlighted the use of mobility data and mechanistic models, our review shows that behavioural integration was strongly contingent on model objectives. By also including statistical models, we further demonstrate that behavioural components were less common in data-driven approaches, a distinction not captured in prior reviews.

To contextualise our findings, we compared our results beyond the Belgian setting with key international modelling efforts, specifically those from SPI-M-O (UK) and the European COVID-19 Forecasting Hub. The UK models, particularly those used for medium-term projections [97], shared notable similarities with the Belgian models in our review. Three of the six models incorporated behavioural components, utilising Google mobility and POLYMOD or CoMix data within an age-structured compartmental model. In contrast, the European Forecasting Hub models, assessed via metadata due to access limitations, showed lower behavioural integration, with only 15 of 67 models explicitly including behavioural components [98]. These models were primarily designed for short-term forecasting in multiple countries, which may have limited their context-specific granularity.

## Conclusions

Overall, our review highlights complementary priorities for integrating behaviour into infectious disease modelling (Table 4). On one hand, there is a need for timely, routine, and disaggregated surveillance and behaviour data to accurately capture disease dynamics. On the other hand, models must be flexible, easy to calibrate, able to integrate diverse data sources, and capable of delivering timely public health insights. Bringing these two sides together also requires robust inference to ensure that models not only fit the data but also generate meaningful and actionable insights. These priorities, distilled into five actionable lessons, resonate beyond the Belgian context and reflect challenges shared across international modelling efforts. Moving forward, stronger collaboration among public health experts, social scientists, data providers, and modellers will be essential to better represent human behaviour in infectious disease models and to enable more effective, evidence-informed public health responses to future outbreaks.

## Supplementary Information

Supplementary Material 1.

## Data Availability

The datasets supporting the conclusions of this article are included within the article (and its supplementary files). All sources analysed in this scoping review are publicly available in the Scopus and PubMed databases.
