# Supplementary material for: A scoping review of COVID-19 modelling studies in Belgium 2020-2024: incorporation of behaviour and lessons learned
Source: Arch Public Health. 2026 May 23;84:160. doi: 10.1186/s13690-026-01959-3 (PMC13383522; doi:10.1186/s13690-026-01959-3)
Supplement: Supplementary file 1 — Supplementary Material 1. [file 13690_2026_1959_MOESM1_ESM.pdf]

Supplementary Material

A Scoping Review of COVID-19 Modelling Studies in Belgium 2020-2024: Incorporation of Behaviour and Lessons Learned

Roel Jude Bagaforo, Marie-Cécile Dupas, Steven Abrams, Simon Dellicour, Niel Hens

S1 Search String

```
(Belgium [tiab] OR belgian [tiab] OR belg* [tiab])
AND (COVID-19 OR SARS-CoV-2 OR coronavirus OR COVID OR 2019-nCoV OR nCoV2019)
AND (compartment* OR contact OR immune OR dynamic* OR short-term OR
long-term OR scenario OR reproductive OR mortality OR virulence OR
transmissibility OR transmission OR hospitalisation OR behav* OR spread)
AND (analys* OR model* OR impact OR effect OR simulat* OR eval*)
```

S2 Model Classes

Table S1: Description of model classes

Model Class	Description and Example
**Mathematical models**	
Compartmental models	Divide the population into compartments and use differential equations to model the flow between them.<br>*Examples:* SIR model and its (compartmental) extensions
Phenomenological models	Capture the shape of the epidemic curve through mathematical functions without mechanistic assumptions about transmission.<br>*Examples:* Logistic growth model, diffusion model
Agent/Individual-based models	Simulate each person (or "agent") and their interactions within a virtual population, naturally accommodating individual-level heterogeneity in behaviour and contact patterns.
Metapopulation models	Extend compartmental models by partitioning the population into discrete geographic patches linked through movement or migration, allowing spatial transmission dynamics to be captured explicitly.
**Statistical models**	
(Mean) Regression models	Describe relationships between a dependent variable (e.g., case counts) and explanatory variables (e.g., mobility, vaccination), focusing on modelling the conditional mean of the outcome.<br>*Examples:* Linear regression, mixed effects models, multivariate regression
Spatial (statistical) models	Account for the spatial autocorrelation and geographic distribution of disease and capture how transmission or risk varies across locations.<br>*Examples:* Endemic-epidemic model, Bayesian hierarchical spatial/spatiotemporal models
Time-series models	Use temporal data to analyse and forecast disease trends over time.<br>*Example:* Autoregressive integrated moving average (ARIMA) model
Machine learning models	Use algorithms that learn patterns from data to make predictions or classifications.<br>*Example:* Long short-term memory neural network
Others	Models not belonging to the previous categories, including those modelling other aspects of the outcome distribution (e.g., shape, location) or specialised models.<br>*Examples:* GAMLSS (generalised additive models for location, scale and shape), Gaussian-Gaussian models, Cox proportional hazards models
**Ensemble models**	Combination of two or more mathematical and/or statistical models to generate a single output, typically to improve predictive performance or capture structural uncertainty across different modelling approaches.<br>*Example:* A weighted combination of a compartmental model and a time-series model to jointly forecast case counts.

S3 Model Objectives

We developed a five-class objective classification to characterise the purposes served by the reviewed models (Table S2).

Table S2: Description of model objective classification

Objective	Definition
Impact of non-pharmaceutical interventions (NPIs)	Models whose objective is to evaluate how non-pharmaceutical interventions, such as lockdowns, social distancing, school closures, or mobility restrictions, affect circulation dynamics or epidemiological outcomes.
Impact of pharmaceutical interventions (PIs)*	Models primarily designed to evaluate the effects of pharmaceutical interventions, including vaccination, antiviral treatments, or prophylaxis, on circulation dynamics or epidemiological outcomes.
Short-term forecasting/ scenario	Models whose primary objective is to predict epidemic dynamics over a short time horizon (less than one month), based on current, recent, or assumed future conditions.
Long-term forecasting/ scenario	Similar to models for "Short-term forecasting/scenario" but for a longer time horizon (a month or more).
Disease metrics	Models used to estimate key disease parameters or metrics, such as the basic or effective reproduction number, fatality ratios, attack rate, excess mortality, or hospitalisation risk.

∗ Under this classification, we examined vaccination-related objectives in greater detail, classifying models according to whether they were used to assess the impact, inform the distribution/allocation/prioritisation, and estimate coverage/threshold/characteristics of vaccination.

The classification is not mutually exclusive, as individual models may address multiple objectives. The following examples illustrate how classification is applied in practice:

- A Poisson regression model examining the association between lockdowns and mortality rate using a stringency index → *Impact of NPIs*
- A compartmental model evaluating the impact of a one-month ban of public gathering through counterfactual projections of cases and deaths in the absence of the intervention → *Impact of NPIs* and *Long-term forecasting/scenario*
- A long short-term memory (LSTM) model projecting case counts over the following week based on current conditions → *Short-term forecasting/scenario*
- A linear mixed model assessing the effect of vaccination while simultaneously estimating excess mortality → *Impact of PIs* and *Disease metrics*
- An agent-based model evaluating the effects of antivirals and school closures through alternative contact scenarios over a three-month horizon, and quantifying associated burden measures to inform intervention prioritisation → *Impact of PIs*, *Impact of NPIs*, *Long-term forecasting/scenario*, and *Disease metrics*

S4 Definition of behaviour and behaviour Change

We defined **behaviour** broadly as any individual or collective action (voluntary or policy-induced) that affects the probability or process of disease transmission. Examples include mask-wearing, hand hygiene, social distancing, public gatherings, and vaccine uptake. Actions like quarantine, isolation, or ICU admission are typically treated as disease states (compartments) that remove individuals from the mixing population. We only classified these as having a behavioural component if the model explicitly included a behavioural factor, such as adherence rate, that governs the flow into these states.

A model may reflect behaviours statically (e.g., a fixed mask-wearing rate). Therefore, we defined **behavioural change** as a dynamic adjustment of these actions or practices, often in response to the epidemic's current state (e.g., rising incidence, changing policies, or risk perception). Examples of dynamic change include immediate or gradual adherence shifts, contact changes, and modified travel patterns.

To illustrate, consider the Force of Infection (FOI) denoted as $\lambda(t)$, i.e.,

$$\lambda(t) = \beta_{base}\,[1 - \tau(t)]\,I(t)/N, \tag{1}$$

where β_{base} is the baseline transmission parameter, $\tau(t)$ is the time-dependent behavioural change parameter, $I(t)$ is the number of infected at time t, and N is the total population and does not depend on time t, implying demographic equilibrium.

- With behaviour only: A constant multiplier ($\tau(t) = \tau$) reflecting a fixed level of contact reduction or mask usage. This model includes behaviour but no behavioural change since τ does not vary.
- With behaviour and behaviour change: $\tau(t)$ is explicitly time-dependent (or state/group-dependent). This model reflects both the behaviour and its dynamic behavioural change.

S5 On the Use of Social Contact Matrices

In this section, we elaborate on the application of social contact matrices observed in the models. Following the social contact hypothesis, transmission is assumed to be directly proportional to the frequency of social encounters. Given age-specific mixing patterns, the transmission rate is defined as:

$$\beta(a, a'; t) = q(t)c(a, a'),$$

where $c(a, a')$ denotes the contact matrix representing the rate at which individuals in age class a interact with those in age class a'. The term $\beta(a, a'; t)$ is the age-specific transmission parameter at time t, with $q(t)$ acting as the proportionality factor (often representing the probability of infection per contact).
To incorporate longitudinal behaviour changes, one can utilise time-varying matrices, $c(a, a'; t)$, such as those derived from the CoMix survey. However, during the early phases of a pandemic, when real-time contact data is often unavailable, models frequently scale pre-pandemic matrices using external proxies:

$$c^{pandemic}(a, a') = \kappa C^{pre-pandemic}(a, a'),$$

$$\beta(a, a'; t) = q(t)\kappa C^{pre-pandemic}(a, a').$$

Here, κ serves as a scaling factor, typically informed by mobility data or policy stringency indices. Furthermore, these matrices can be stratified to differentiate between disease states, such as asymptomatic versus symptomatic individuals, while maintaining the underlying contact structure:

$$\beta^{symp}(a, a'; t) = q(t)c^{symp}(a, a'); \quad \beta^{asymp}(a, a'; t) = q(t)c^{asymp}(a, a').$$

S6 PRISMA-ScR Checklist

Preferred Reporting Items for Systematic reviews and Meta-Analyses extension for Scoping Reviews (PRISMA-ScR) Checklist

SECTION	ITEM	PRISMA-ScR CHECKLIST ITEM	REPORTED ON PAGE #
**TITLE**			
Title	1	Identify the report as a scoping review.	1
**ABSTRACT**			
Structured summary	2	Provide a structured summary that includes (as applicable): background, objectives, eligibility criteria, sources of evidence, charting methods, results, and conclusions that relate to the review questions and objectives.	2
**INTRODUCTION**			
Rationale	3	Describe the rationale for the review in the context of what is already known. Explain why the review questions/objectives lend themselves to a scoping review approach.	2-3
Objectives	4	Provide an explicit statement of the questions and objectives being addressed with reference to their key elements (e.g., population or participants, concepts, and context) or other relevant key elements used to conceptualize the review questions and/or objectives.	3 and 7
**METHODS**			
Protocol and registration	5	Indicate whether a review protocol exists; state if and where it can be accessed (e.g., a Web address); and if available, provide registration information, including the registration number.	No protocol was registered for this scoping review. However, an internal protocol was developed and followed to guide the review process. See pg 4-6
Eligibility criteria	6	Specify characteristics of the sources of evidence used as eligibility criteria (e.g., years considered, language, and publication status), and provide a rationale.	5-6
Information sources*	7	Describe all information sources in the search (e.g., databases with dates of coverage and contact with authors to identify additional sources), as well as the date the most recent search was executed.	4
Search	8	Present the full electronic search strategy for at least 1 database, including any limits used, such that it could be repeated.	4-5 and S1
Selection of sources of evidence†	9	State the process for selecting sources of evidence (i.e., screening and eligibility) included in the scoping review.	4-6
Data charting process‡	10	Describe the methods of charting data from the included sources of evidence (e.g., calibrated forms or forms that have been tested by the team before their use, and whether data charting was done independently or in duplicate) and any processes for obtaining and confirming data from investigators.	6-7
Data items	11	List and define all variables for which data were sought and any assumptions and simplifications made.	6-7

St. Michael's
Inspired Care.
Inspiring Science.

1

SECTION	ITEM	PRISMA-ScR CHECKLIST ITEM	REPORTED ON PAGE #
Critical appraisal of individual sources of evidence§	12	If done, provide a rationale for conducting a critical appraisal of included sources of evidence; describe the methods used and how this information was used in any data synthesis (if appropriate).	6-7
Synthesis of results	13	Describe the methods of handling and summarizing the data that were charted.	7
**RESULTS**			
Selection of sources of evidence	14	Give numbers of sources of evidence screened, assessed for eligibility, and included in the review, with reasons for exclusions at each stage, ideally using a flow diagram.	7-8
Characteristics of sources of evidence	15	For each source of evidence, present characteristics for which data were charted and provide the citations.	8-16
Critical appraisal within sources of evidence	16	If done, present data on critical appraisal of included sources of evidence (see item 12).	8-16
Results of individual sources of evidence	17	For each included source of evidence, present the relevant data that were charted that relate to the review questions and objectives.	8-16
Synthesis of results	18	Summarize and/or present the charting results as they relate to the review questions and objectives.	8-16
**DISCUSSION**			
Summary of evidence	19	Summarize the main results (including an overview of concepts, themes, and types of evidence available), link to the review questions and objectives, and consider the relevance to key groups.	16-20
Limitations	20	Discuss the limitations of the scoping review process.	18-20
Conclusions	21	Provide a general interpretation of the results with respect to the review questions and objectives, as well as potential implications and/or next steps.	20
**FUNDING**			
Funding	22	Describe sources of funding for the included sources of evidence, as well as sources of funding for the scoping review. Describe the role of the funders of the scoping review.	21

JBI = Joanna Briggs Institute; PRISMA-ScR = Preferred Reporting Items for Systematic reviews and Meta-Analyses extension for Scoping Reviews.
* Where *sources of evidence* (see second footnote) are compiled from, such as bibliographic databases, social media platforms, and Web sites.
† A more inclusive/heterogeneous term used to account for the different types of evidence or data sources (e.g., quantitative and/or qualitative research, expert opinion, and policy documents) that may be eligible in a scoping review as opposed to only studies. This is not to be confused with *information sources* (see first footnote).
‡ The frameworks by Arksey and O'Malley (6) and Levac and colleagues (7) and the JBI guidance (4, 5) refer to the process of data extraction in a scoping review as data charting.
§ The process of systematically examining research evidence to assess its validity, results, and relevance before using it to inform a decision. This term is used for items 12 and 16 instead of "risk of bias" (which is more applicable to systematic reviews of interventions) to include and acknowledge the various sources of evidence that may be used in a scoping review (e.g., quantitative and/or qualitative research, expert opinion, and policy document).

From: Tricco AC, Lillie E, Zarin W, O'Brien KK, Colquhoun H, Levac D, et al. PRISMA Extension for Scoping Reviews (PRISMAScR): Checklist and Explanation. Ann Intern Med. 2018;169:467–473. doi: 10.7326/M18-0850.
